# Linking Maternal Educational Involvement to Early Adolescent Academic Adjustment: The Moderating Role of Maternal Authoritativeness

**DOI:** 10.3390/bs16071147

**Published:** 2026-07-08

**Authors:** Sze Wan Virginia Lam, Chun Bun Lam

**Affiliations:** B2-1/F-28, Department of Early Childhood Education, Faculty of Education and Human Development, The Education University of Hong Kong, Hong Kong, China; hi.missvg@gmail.com

**Keywords:** parental educational involvement, home- and school-based involvement, adolescent academic adjustment, parenting styles, Chinese families

## Abstract

This cross-sectional study examined the association of maternal educational involvement with early adolescent academic adjustment and tested maternal authoritativeness as a moderator. Participants were 665 senior elementary school students (52% were boys; mean age = 10.54 years; age range = 9.08–14.83) from Hong Kong, China. Using paper-and-pencil questionnaires, students rated their mothers’ home-based involvement (i.e., support and encouragement, expectations and attitudes, and rules and monitoring) and school-based involvement (i.e., communication with teachers and participation in school events) as well as their mothers’ overall authoritativeness. Students also rated their own academic adjustment (i.e., motivation, skills, and competence). Hierarchical regressions indicated that maternal support and encouragement were uniquely linked to early adolescent academic skills, whereas maternal rules and monitoring were uniquely linked to early adolescent academic motivation and competence. Moreover, especially for students with authoritative mothers, maternal expectations and attitudes were uniquely linked to academic motivation, skills, and competence, and maternal communication with teachers was uniquely linked to early adolescent academic skills and competence. Findings highlighted the importance of conceptualizing parental educational involvement and adolescent academic adjustment as multidimensional constructs and differentiating between parenting practices versus parenting styles. Practically, interventionists and policy makers may consider targeting both maternal educational involvement and maternal authoritativeness as possible ways to promote early adolescent academic adjustment.

## 1. Introduction

Parental educational involvement has long been theorized to promote youth academic development (Boonk et al., 2018). However, although some studies found parental educational involvement to be positively linked to youth academic adjustment (Cheung & McBride-Chang, 2008; Quilliams & Beran, 2009; Schmid & Garrels, 2021; Stright & Yeo, 2014; Wang et al., 2014), others reported null and even negative findings (Driessen et al., 2005; Fan et al., 2012; O’Sullivan et al., 2014; Pomerantz et al., 2007; Silinskas et al., 2015; Zhu et al., 2023). These inconsistent results may be due to the focus on different forms of parental involvement and the use of univariate versus multivariate analyses (Kim, 2022) as well as a lack of differentiation between specific parenting practices versus overall parenting styles (Xia et al., 2020; Zong et al., 2018). Grounded in the notions that parental involvement is multidimensional (Fantuzzo et al., 2000) and that overall parenting styles often qualify the impact of specific parenting practices (Darling & Steinberg, 1993), the present study tested whether different forms of maternal educational involvement, including support and encouragement, expectations and attitudes, rules and monitoring, communication with teachers, and participation in school events, were uniquely linked to early adolescent academic adjustment, and whether these links were moderated by maternal authoritativeness, an overall index of parenting style.

We focused on three distinct but related academic outcomes—motivation, skills, and competence—as they roughly captured the will, the skill, and the performance dimensions of youth academic development (Arnold & Rowaan, 2014; Rose et al., 2019). Indeed, researchers have increasingly defined academic adjustment as more than having good grades (i.e., showing good performance). Also important are intrinsic motivation to learn (i.e., having the will) and self-regulatory and knowledge acquisition abilities that make learning effective (i.e., having the skills) (Costa et al., 2024). We conducted this study with a sample of senior elementary school students from Hong Kong, China, given that psychological research has predominantly relied on European and European American samples (Reyna et al., 2023) and that Chinese societies place an exceptionally strong emphasis on youth academic achievement (Zhang & Csizmadia, 2024). Furthermore, we examined maternal (rather than paternal) involvement, as mothers spend more time with children and adolescents than do fathers (Lam et al., 2012; Lam & McHale, 2015). Moreover, in modern Chinese society, mothers more often play the role of “strict academic supervisors,” whereas fathers more often play the role of “friendly leisure companions” (Luo et al., 2021; Tam, 2009).

### 1.1. Parental Educational Involvement as a Multidimensional Construct

Numerous theoretical frameworks have conceptualized parental educational involvement as a multidimensional construct, classifying parental educational involvement into distinct but correlated aspects that may be uniquely important to youth academic development (Fantuzzo et al., 2000). No two theoretical frameworks are exactly the same, but parental educational involvement is typically classified into home- versus school-based involvement (Castro et al., 2015): Home-based involvement includes parent–child interactions that take place outside of school, such as parental provision of support and encouragement, parental communication about their expectations and attitudes about schoolwork, and parental establishment of rules and monitoring to ensure that their children complete their homework and study for tests and examinations. Meanwhile, school-based involvement includes parent–child interactions that take place in school and home-school collaborations, such as communication with teachers and participating in and volunteering for school events.

However, a multidimensional conceptualization of parental educational involvement is not always reflected in empirical investigations, with some research focusing only on home-based involvement (Chen & Ho, 2012; Gonida & Cortina, 2014; Gonzalez-Pienda et al., 2002; Li et al., 2019) and some focusing only on school-based involvement (Gutman & Eccles, 1999; Simpkins et al., 2006). A smaller body of work covered both forms of involvement, but this work tended to cover multiple aspects of home-based involvement but only one single aspect of school-based involvement (Altschul, 2011; Ceballo et al., 2014; Hong & Ho, 2005). Additionally, possibly due to the focus on different forms of parental involvement and the reliance on univariate versus multivariate analyses (Kim, 2022), some studies documented positive associations between parental involvement and youth adjustment (Cheung & McBride-Chang, 2008; Quilliams & Beran, 2009; Stright & Yeo, 2014; Wang et al., 2014), whereas others reported null (O’Sullivan et al., 2014; Pomerantz et al., 2007) and even negative associations (Driessen et al., 2005; Fan et al., 2012; Silinskas et al., 2015; Zhu et al., 2023).

One exception was Catsambis’s (2001) study, which examined the unique impact of multiple aspects of home-based involvement and multiple aspects of school-based involvement in the high school years. Their results indicated that both home-based (i.e., support and encouragement and rules and monitoring) and school-based involvement (i.e., participation in school events) of parents were uniquely linked to their adolescents’ performance in math, science, and reading. Expanding upon this work, we examined the unique impact of three aspects of home-based involvement (i.e., support and encouragement, expectations and attitudes, and rules and monitoring; Cheung & McBride-Chang, 2008) and two aspects of school-based involvement (i.e., communication with teachers and participation in school-based events; Manz et al., 2004). Based on a multidimensional conceptualization of parental educational involvement (Fantuzzo et al., 2000), we expected that both home- and school-based aspects of maternal educational involvement would be uniquely linked to early adolescent academic adjustment.

### 1.2. Parenting Styles as Emotional Contexts

Another possible explanation for the inconsistent findings on parental involvement and youth adjustment concerns the potential moderating role of parenting styles. In contrast to specific parenting practices (such as parental educational involvement), parenting styles represent a broader concept that portrays the overall emotional climate of the parent–child relationship (Masud et al., 2015). Baumrind (1971), for example, argued that parenting styles communicate the general attitudes parents hold toward their children, with authoritarian parents expressing high expectations for but low warmth toward their children, permissive parents expressing low expectations but high warmth, and authoritative parents being high on both expectations and warmth. Darling and Steinberg (1993) further proposed that the implications of parenting practices may vary depending on the emotional climate in which these practices operate. As parenting styles set the overarching tone of parent–child relationships, they may affect children’s willingness to cooperate with their parents’ socialization efforts. Children with less authoritative parents may perceive their parents’ behaviors as intrusive or controlling, and thus are more resistant to parental socialization. In contrast, children with more authoritative parents may interpret their parents’ behaviors as driven by reasonable expectations and genuine concern, and thus are more susceptible to parental socialization (Pomerantz et al., 2007).

A number of studies tested parenting styles as a moderator in the association between parental educational involvement and youth academic adjustment. For example, in Simpkins et al.’s (2006) study with kindergarten-aged children in the U.S., school-based parental involvement (i.e., home-school communication) was more strongly associated with child performance in math and reading tests, especially when the mothers exhibited more warmth in daily interactions. Similarly, in Stright and Yeo’s (2014) study with school-aged children from Singapore, maternal school-based parental involvement (i.e., participation in school events) was associated with child performance in school exams on English, math, and science, but only for children with warm mothers. Moreover, in Wang et al.’s (2014) study with American adolescents, parental home-based parental involvement (i.e., rules and monitoring) was more strongly correlated with child grade point averages, especially when children perceived their parents to be warmer. More recent studies focused on academic outcomes other than the performance of children. For example, in Xia et al.’s (2020) study with Chinese kindergarten-aged children, school-based parental involvement in general was associated with child school readiness (i.e., the skills to perform well in school), but only for children with more authoritative mothers. Moreover, in Zong et al.’s (2018) study with Chinese school-aged children, home-based parental involvement in general was associated with child performance goal orientation to learning (i.e., the will to perform well in school), but only for children with less controlling parents.

These findings suggested that parenting styles (especially warmth) may operate as a moderator. However, warmth is only one constituting component of parental authoritativeness—a theoretically integrative and widely studied parenting style that simultaneously encompasses high warmth and high expectations (Baumrind, 1971). Indeed, relatively little is known about the potential moderating role of parental authoritativeness in the links of different forms of parental educational involvement with different child academic outcomes, especially those that are less performance-based, such as the will and skills to do well in school (Arnold & Rowaan, 2014; Rose et al., 2019). Guided by the view that parenting styles may define the emotional climate of the embedding parent–child relationship and modulate the impact of specific parenting practices (Darling & Steinberg, 1993), we expected that maternal educational involvement would more strongly linked to the academic adjustment of early adolescents with more authoritative mothers.

### 1.3. The Present Study

To recap, using cross-sectional questionnaire data from Chinese elementary school students, we examined whether different aspects of home- and school-based maternal educational involvement were uniquely associated with early adolescent academic motivation, skills, and competence, and whether these associations were moderated by maternal authoritativeness. Given the exceptionally high cultural emphasis on academic achievement (Zhang & Csizmadia, 2024) and the “scripted” roles of mothers as academic supervisors in Chinese societies (Luo et al., 2021; Tam, 2009), we hypothesized that maternal support and encouragement, expectations and attitudes, rules and monitoring, communication with teachers, and participation in school events would be positively linked with early adolescent motivation, skills, competence. Moreover, we hypothesized that the associations to be stronger among families with more authoritative mothers. Given prior research showing that girls, older students, and students from higher socioeconomic backgrounds are academically better adjusted than boys, younger students, and students from lower socioeconomic backgrounds, respectively (Peng & Kievit, 2020), we controlled for adolescent gender and age and maternal education.

## 2. Materials and Methods

### 2.1. Participants and Procedures

Participants were 665 fourth to sixth graders from five elementary schools in Hong Kong, China. We used a stratified sampling approach to ensure that families from diverse socioeconomic backgrounds were recruited: Hong Kong has 18 geographic districts. Based on the average median monthly income of all households in Hong, we classified these districts into high, middle, and low socioeconomic districts. We then randomly called elementary schools located in these districts using publicly available contact information, until two small- to medium-sized schools, one large-sized school, and two small- to medium-sized schools from high, middle, and low socioeconomic districts agreed to participate, respectively. Invitation letters and consent forms were sent home to the fourth to sixth graders through these five schools. Of the 1465 students invited, 46% (*N* = 676) returned consent forms with both parental and adolescent signatures and eventually provided data. Under the supervision of trained administrators, students completed questionnaires on their family relationships and academic adjustment during school hours in sessions of 25–35 min. Each student received a small stationery gift of HK$6 (about US$1), upon the completion of the questionnaire. This study was conducted in accordance with the Declaration of Helsinki. The procedures were approved by Faculty Research and Higher Degree Committee, Faculty of Education and Human Development, The Education University of Hong Kong.

After excluding the data from 11 students who were not living with their mothers, who provided unusual responses, such as “zigzag” patterns (e.g., 1, 2, 3, 4, 5, 1, 4, 3, 2, 1 across consecutive items) or straight-line patterns (e.g., all 1 s or all 5 s on an entire page), or who missed more than 50% of the questionnaires, we based our analyses on 665 students. Of these 665 students, 40%, 38%, and 22% were studying in high, middle, and low socioeconomic districts, respectively. According to Census and Statistics Department (2026), 48%, 37%, and 17% of Hong Kong households lived in high, middle, and low socioeconomic districts, respectively. Therefore, our sample appeared to be of slightly higher socioeconomic status compared to the general population.

Mothers provided demographic information, including adolescent gender and age and maternal education. Students’ mean age was 10.54 years old (*SD* = 0.90; range = 9.08–14.83). We described our sample as early adolescents, given that early adolescence is widely defined as the developmental period between 10 and 13 years (American Psychological Association, 2002)—sometimes stretching from the beginning of puberty (which can occur as young as 8–9 years) to about 14 years (Sawyer et al., 2018). The numbers of boys (*n* = 346, 52%) and girls (*n* = 324, 48%) were roughly equal.

### 2.2. Measures

Item ratings were averaged for each measure, with higher scores indicating higher levels of the construct.

*Maternal home-based involvement* was measured using the Maternal Academic Practice Scale (Cheung & McBride-Chang, 2008). On a 5-point scale (1 = *Strongly Disagree*; 5 = *Strongly Agree*), students rated how often their mothers were involved in their education at home. This measure included three subscales, namely support and encouragement (e.g., “My mother praises me when I work hard”; 5 items; α = 0.82), expectations and attitudes (e.g., “My mother says that working hard grants a bright future”; 3 items; α = 0.57), and rules and surveillance (e.g., “My mother does not allow me to watch TV until I finish homework”; 4 items; α = 0.60). We referred to the subscale of rules and surveillance as rules and *monitoring*, as surveillance typically indicates intrusive, unwanted, and covert monitoring, which may not accurately reflect the construct assessed here. In fact, in Chinese communities, many adolescents view parental monitoring, especially in the academic domain, as legitimate and beneficial, even though it sometimes may generate negative emotions or even parent–child conflict (Leung & Shek, 2020; Zhang & Csizmadia, 2024). As the subscale of expectations and attitudes showed exceptionally low reliability, we followed Tavakol and Dennick’s (2011) recommendations and examined the inter-item and item-total correlations. One item (“My mother does not care about my exam results”) had relatively low correlations with the other two items in the subscale (*r*s = 0.23 and 0.22) and with the total score (*r* = 0.26). In fact, this item was the only item that was negatively worded and reversely coded in the subscale. Therefore, we removed it to improve the Cronbach alpha of the subscale of expectations and attitudes to α = 0.65.

*Maternal school-based involvement* was measured using two subscales from the Family Involvement Questionnaire (Manz et al., 2004): home-school communication (e.g., “My mother talks to my teachers about work I should practice at home”; 7 items; α = 0.87) and participation in school-based activities (e.g., “My mother volunteers in the school”; 7 items; α = 0.83). On a 4-point scale (1 = *Rarely*; 4 = *Always*), students rated how often their mothers were involved in their education in school. Each of the two subscales originally included 13 items. However, due to time constraints, only the seven items with the highest factor loadings in each subscale documented in Manz et al.’s (2004) study were used in the present study. This item-selection approach is a well-established practice for developing briefer yet reliable and valid measures (Boateng et al., 2018). Moreover, research on scale development with children and adolescents commonly considers six items per subscale to be sufficient for achieving acceptable reliability and validity (e.g., Harter, 2012; Spence, 1998). Therefore, we retained one additional item (seven items in total per subscale) as a “safety margin” to ensure robust internal consistency.

*Maternal authoritativeness* was measured using the 15-item authoritative parenting subscale of the Parenting Styles and Dimensions Questionnaire (Robinson et al., 2001). On a 5-point scale (1 = *Never*; 5 = *Always*), students rated how often their mothers displayed high expectations for and high warmth toward them (e.g., “My mother gives me reasons why rules should be obeyed,” “My mother has warm and intimate times together with me”; α = 0.90).

*Early adolescent academic motivation* was measured using the 8-item learning goal orientation subscale from the Goal Orientation Scale (Button et al., 1996). On a 5-point scale (1 = *Strongly Disagree*; 5 = *Strongly Agree*), students rated their motivation to work hard and complete tasks in order to learn new things (e.g., “I prefer to work on tasks that force me to learn new things”; α = 0.83).

*Early adolescent academic skills* were measured using the 7-item Good Student Scale (Gesten, 1976). On a 5-point scale (1 = *Strongly Disagree*; 5 = *Strongly Agree*), students rated their abilities to focus on school work, function as a good learner, and succeed in school (e.g., “I work well without adult support”; α = 0.77).

*Early adolescent academic competence* was measured using the 6-item scholastic competence subscale from Harter’s (2012) Self-perception Profile for Children. Using a structured alternative format, students indicated, on a 4-point scale, which type of youth they could better relate to in the aspect of academic adjustment (e.g., “Some kids have trouble figuring out the answers in school BUT other kids almost always can figure out the answers”; α = 0.70). Students first decided which description was more like themselves and then selected whether it was “*Really True for Me*” or “*Sort of True for Me.*”

Demographic characteristics, including early adolescent gender and age and maternal education, were provided by students’ mothers. Mothers rated their education by indicating the highest education level attained, ranging from “Never received any education” to “Completed a Doctor of Philosophy degree”.

## 3. Results

Table 1 presents the descriptive statistics of variables. In educational and psychological research, bivariate correlations (*r*s) of about 0.10, 0.30, and 0.50 are commonly interpreted as small, medium, and large effect sizes, respectively (Cohen, 1988; Funder & Ozer, 2019). The five forms of maternal educational involvement were modestly to strongly correlated with one another (*r*s = 0.09–0.61), with the two school-based involvement most strongly correlated with each other (*r* = 0.61), and expectations and attitudes and participation in school events most weakly correlated (*r* = 0.09). Moreover, all five forms of maternal educational involvement were modestly to strongly correlated with the three adolescent academic outcomes (*r*s = 0.09–0.45), with maternal support and encouragement generally showing the strongest correlations (*r*s = 0.23–0.41) and maternal participation in school events generally showing the weakest correlations (*r*s = 0.13–0.16).

The skewness values of substantive variables ranged from −1.05 to 0.70 and the kurtosis values ranged from −0.73 to 1.04, falling within the acceptable ranges (skewness between −2 and +2; kurtosis between −7 and +7), suggesting that the assumption of normality was not violated (Hair et al., 2019). Moreover, the variance inflation factors (VIF) values of predictor variables ranged from 1.42 to 3.02, well below the cutoff of 5, indicating that multicollinearity was not a major concern. Less than 2% of the data were missing. Little’s MCAR test further indicated that the data were missing completely at random, *χ*^2^(71) = 65.03, nonsignificant (n.s.). Therefore, the pairwise deletion method was used to handle our missing data (Peugh & Enders, 2004).

Following Frazier et al.’s (2004) approach, we ran hierarchical regression analyses separately for each of the three outcome variables. In Step 1, we entered demographic variables, including early adolescent gender and age and maternal education. In Step 2, we entered the standardized scores of maternal authoritativeness, support and encouragement, expectations and attitudes, rules and monitoring, home-school communication, and participation in school events. In Step 3, we entered the two-way interactions between the standardized scores of maternal authoritativeness and the five aspects of maternal educational involvement. The patterns of significant interactions were graphed by plotting the predicted values of the outcome variables based on the predictor variable, when maternal authoritativeness was high (+1 *SD*) and low (−1 *SD*), respectively.

Nonsignificant interaction terms were removed from the final models, as retaining them would introduce multicollinearity and change the interpretation of the lower-order main effect terms (Aiken & West, 1991; Cohen et al., 2003; Jaccard & Turrisi, 2003). As interaction terms were created by multiplying the main effect terms, nonsignificant interactions were highly correlated with their constituent main effects. Therefore, retaining nonsignificant interactions would unnecessarily inflate standard errors. Moreover, nonsignificant interactions redefine the lower-order main effects by making them represent the association between the predictor and outcome variables when the moderator variable is at zero (rather than at the sample mean). Therefore, retaining nonsignificant interaction terms might lead to misleading conclusions. While some researchers prefer retaining all hypothesized interactions for full transparency, we opted to trim them to ensure more stable models and more interpretable estimates. As three regression models were tested, we applied a Bonferroni correction and set the adjusted significance level at *p* = 0.05/3 ≈ 0.017 to preempt the inflation of Type I errors due to multiple testing.

Table 2 presents the standardized coefficients of the regression models. Controlling for demographic variables, maternal authoritativeness, and other aspects of maternal educational involvement, Step 2 showed that maternal support and encouragement were uniquely linked to adolescent academic skills, whereas maternal rules and monitoring were uniquely linked to adolescent academic motivation and competence. Maternal expectations and attitudes were linked to adolescent academic motivation and skills, but these links were qualified by the two-way interactions entered in Step 3, as described below. Maternal participation in school events was not a significant predictor in any models.

**Figure 1:**
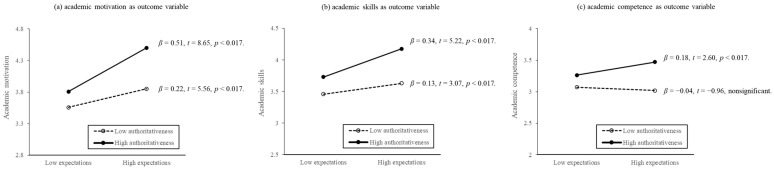
Associations between maternal expectations and attitudes and early adolescent academic motivation, skills, and competence by levels of maternal authoritativeness.

**Figure 2:**
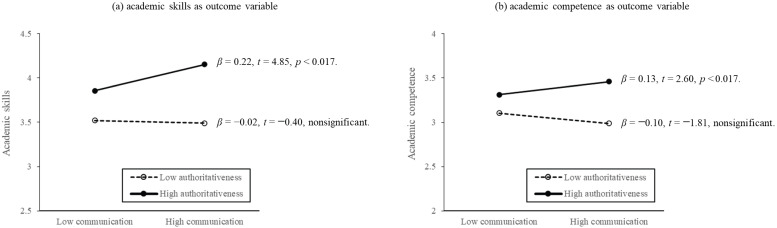
Associations between maternal communication with teachers and early adolescent academic motivation, skills, and competence by levels of maternal authoritativeness.

Step 3 detected five significant interactions between maternal authoritativeness and two forms of maternal educational involvement: expectations and attitudes and communication with teachers. As noted above, the associations of maternal expectations and attitudes with academic motivation (Figure 1a) and skills (Figure 1b) were stronger for early adolescents with more authoritative mothers (*β*s = 0.34–0.51, *p*s < 0.017) than for those with less authoritative mothers (*β*s = 0.13–0.22, *p*s < 0.017). Meanwhile, the association of maternal expectations and attitudes with academic competence (Figure 1c) was only significant for early adolescents with more authoritative mothers (*β* = 0.18, *p* < 0.017) but not for those with less authoritative mothers (*β* = −0.04, n.s.). Finally, the associations of maternal communication with teachers with academic skills (Figure 2a) and competence (Figure 2b) were significant for early adolescents with more authoritative mothers (*β*s = 0.13–0.22, *p*s < 0.017) but not for those with less authoritative mothers (*β*s = −0.10–0.02, n.s.).

In educational and psychological research, unique variance explained (Δ*R*^2^) of about 1–4%, 5–9%, and 10% or more are commonly interpreted as small, medium, and large effect sizes, respectively (Cohen, 1988; Funder & Ozer, 2019). In this study, Step 2 (the main effect block) explained an additional 9% to 31% of variance in measures of early adolescent academic adjustment, indicating medium to large effect sizes. In contrast, Step 3 (the significant interaction effect block) explained an additional 2% to 3% of variance in early adolescent academic adjustment, indicating small effect sizes.

## 4. Discussion

This study showed that maternal support and encouragement were uniquely linked to early adolescent academic skills and that maternal rules and monitoring were uniquely linked to early adolescent academic motivation and competence. Moreover, especially for early adolescents with authoritative mothers, maternal expectations and attitudes were uniquely linked to early adolescent academic motivation, skills, and competence, whereas maternal communication with teachers was uniquely linked to early adolescent academic skills and competence.

### 4.1. Home- and School-Based Involvement Explained Unique Variance

Parental educational involvement was linked to youth academic outcomes in some studies (Cheung & McBride-Chang, 2008; Quilliams & Beran, 2009; Schmid & Garrels, 2021; Stright & Yeo, 2014; Wang et al., 2014), but not others (Driessen et al., 2005; Fan et al., 2012; O’Sullivan et al., 2014; Pomerantz et al., 2007; Silinskas et al., 2015; Zhu et al., 2023), potentially due to the focus on different forms of parental involvement and the reliance on univariate versus multivariate analyses in different studies (Kim, 2022). Consistent with such views, although all predictor variables were significantly correlated with all outcome variables in our *univariate* analyses, only maternal support and encouragement, expectations and attitudes, rules and monitoring, and communication with teachers were uniquely linked to specific adolescent academic outcomes in our *multivariate* analyses.

Maternal rules and monitoring were linked to both adolescent academic motivation and skills, a finding inconsistent with prior meta-analyses showing that the association of parental rules and monitoring with adolescent academic achievement was weaker compared to other forms of parental involvement (Castro et al., 2015; Kim, 2022). However, given that these meta-analyses were based mostly on European and European American families but that our study was based on Chinese families, cultural differences between Western and Eastern contexts could be a contributing factor. Previous research has indicated that Chinese parents’ intensive arrangement and supervision of their youth’s activities, particularly in the academic domain, are often perceived by both parents and their youth to be necessary and beneficial (Leung & Shek, 2020; Zhang & Csizmadia, 2024). Therefore, Chinese youth may be more accepting of and susceptible to their parents’ imposition of daily life structures. It is important to note that our study was solely based on one cultural group; a cultural interpretation of our findings was not directly tested and remained speculative. Future researchers should use culture-comparative designs to examine whether parental rules and monitoring are more strongly correlated with youth outcomes in the West than in the East. Future researchers should also use cultural homogeneous designs to examine whether the strength of the correlation between parental rules and monitoring and youth academic outcomes varies as a function of individual endorsement cultural values, such as filial piety values in Chinese communities.

One study with adolescents showed that, controlling for parental home-based involvement and parental communication with teachers, parental participation in school events was uniquely and positively associated with adolescent performance in tests of math, science, and reading (Catsambis, 2001). In contrast, our study showed that maternal participation in school events was not uniquely linked to early adolescent academic motivation, skills, or competence. It is hard to produce a definite explanation, especially given that our study did not assess adolescent performance in school exams, as in Catsambis’s (2001) study. However, our study did control for maternal overall parenting styles, an important construct that sets the overarching tone of parent–child relationships (Masud et al., 2015). Indeed, even controlling for specific educational involvement, maternal authoritativeness remained uniquely linked to all three early adolescent academic outcomes. Additionally, as youth grow older, they may have lower expectations for their parents’ direct participation in school events, which might reduce the unique contribution of this form of parental involvement to youth academic adjustment. Furthermore, during adolescence, when youth are increasingly striving for autonomy and independence, being seen with their parents in school or having parents who are “overly” involved may be embarrassing or undesirable (Scholte & Van Aken, 2020). An important direction for future research is to examine whether and how developmental stage moderates the role of parental participation in school events in understanding both youth self-perceived academic adjustment and objective academic performance, above and beyond parenting styles and other forms of parental educational involvement.

In keeping with the view that academic adjustment can be defined from the will, the skill, and the performance dimensions (Arnold & Rowaan, 2014; Costa et al., 2024; Rose et al., 2019), academic motivation, skills, and competence were modestly to strongly correlated with one another in our data. Moreover, different forms of maternal educational involvement were differentially associated with different outcomes (e.g., support and encouragement were associated with skills, whereas rules and monitoring were associated with motivation and competence). As our study was based on cross-sectional data from only one sample, additional efforts should be directed at replicating these differential associations. However, our study did highlight the value of examining academic adjustment as a multidimensional construct rather than a single global index—as we did for parental educational involvement.

### 4.2. Maternal Authoritativeness as a Moderator

Prior studies have shown that parental warmth, a constituting component of overall parenting styles (Baumrind, 1971), might moderate the association of parental educational involvement and youth performance in tests and exams (Simpkins et al., 2006; Stright & Yeo, 2014; Wang et al., 2014). Prior studies have also shown that parental authoritativeness and parental psychological control might moderate the associations of parental educational involvement and youth academic skills and motivation (Zong et al., 2018; Xia et al., 2020). Expanding upon this work, our study demonstrated that maternal authoritativeness moderated the associations of maternal expectations and attitudes with early adolescent academic motivation, skills, and competence and the associations of maternal communication with teachers with adolescent academic skills and competence. The unique contribution of our study rested in our focus on multiple aspects of parental involvement *and* multiple aspects of adolescent academic adjustment and our differentiation between parenting practices and parenting styles. On the most general level, our findings suggested youth may be more susceptible to parents’ socialization efforts when they perceive these efforts to be more reasonable and more caring (Pomerantz et al., 2007). Importantly, our findings were based on cross-sectional data from only one sample. Replications of our findings using longitudinal designs and additional samples are needed. Further efforts should also be directed at examining whether parental involvement embedded in a more authoritative parent–child relational context is actually perceived by youth to be more legitimate and more earnest.

Our findings had important theoretical and practical implicatons. Theoretically, when studying the implications of specific parenting behaviors, researchers should consider the emotional climate of parent–child relationships, potentially indicated by overall parenting styles (Darling & Steinberg, 1993). Practically, when getting involved in their early adolescents’ education, either at home or in school, parents may attend to the overarching parenting styles in which these parenting practices operate. Meanwhile, when designing campaigns to promote early adolescents’ academic adjustment, interventionists and policy makers may consider targeting both maternal authoritativeness and involvement.

### 4.3. Limitations

This study was not without limitation. First, although a stratified sampling approach was used to recruit families from different socioeconomic backgrounds, the final sample appeared to be of higher socioeconomic status compared to the Hong Kong population, possibly reflecting that individuals of higher socioeconomic status are more willing to participate in academic research (Groves & Peytcheva, 2008). The generalizability of our findings awaits further examination, particularly with more regionally representative samples. Second, our cross-sectional design did not allow us to draw firm conclusions about causation. Randomized experimental designs are needed to disentangle the causal relationships between parental educational involvement and youth academic adjustment. Third, both our predictor and outcome variables were based on early adolescent self-reports, which introduced shared method variance and self-perception bias into our findings. Future research should address these issues using multi-informant designs and observation- and performance-based measures (e.g., linking parental reports of involvement with youth behaviors during independent observations and to youth performance in actual tests).

Fourth, the measures of expectations and attitudes as well as rules and monitoring showed relatively low internal consistency. In particular, the reliability of expectations and attitudes measure remained marginal even after removing a reverse-coded item (α = 0.65), and that of the rules and monitoring measure was even lower (α = 0.60). As measures with low reliability tend to increase measurement errors and attenuate observed associations (Nimon et al., 2012), we might have underestimated the strength of some relationships. Future research should employ measures with stronger psychometric properties to assess parental educational involvement. Fifth, due to time constraints, we were not able to use the full 13-item subscales of home-school communication and participation in school-based activities (Manz et al., 2004). Instead, we selected the seven items with the highest factor loadings in each subscale (Boateng et al., 2018). Although our shortened versions showed acceptable reliabilities (αs > 0.80), future researchers should use the full versions to improve the robustness of their findings. Sixth, as mothers often serve as the primary caregivers (Lam et al., 2012; Lam & McHale, 2015) and academic supervisors (Luo et al., 2021; Tam, 2009) of youth, we only focused on maternal educational involvement. However, fathers are increasingly involved in childrearing; future studies should also examine whether paternal involvement is linked to child outcomes in similar ways, and whether parental gender moderates the relationship between parental involvement and child outcomes. Additionally, due to the increasingly varied family compositions of modern society, the roles of other caregivers, such as grandparents and older siblings, should be considered.

Seventh, the primary goal of this study was to examine the substantive interrelationships among maternal authoritativeness, maternal educational involvement, and early adolescent academic adjustment, rather than to evaluate the psychometric properties of the measures. Therefore, our recruitment procedures were not designed to test for measurement equivalence across demographic groups (e.g., age or socioeconomic groups). Future studies should be designed with sufficiently large and balanced subgroups to examine measurement invariance across demographic characteristics. Establishing whether constructs function similarly across demographic groups would further strengthen the validity of our findings. Relatedly, although our sample fell within the age range of early adolescence (American Psychological Association, 2002; Sawyer et al., 2018), the age distribution was relatively wide. Whether and how age moderates the associations between different forms of maternal educational involvement with youth academic adjustment warrants examination, especially with samples that have sufficiently large and balanced age groups. Eighth, although some interaction terms were significant, their patterns consistent with our hypotheses, they explained only an additional 2% to 3% of the variance in early adolescent academic adjustment, indicating that maternal parenting styles and parenting practices represented only part of the story. Like other domains of adjustment, youth academic adjustment is likely affected by the joint function of intrapersonal and interpersonal factors, which in turn are nested within the family, school, and community contexts (Peng & Kievit, 2020). Future research should examine how these multi-layered factors may interact to shape early adolescent development.

Finally, at a conceptual level, parenting styles can be distinguished from parenting practices. However, in reality, parenting styles and parenting practices are likely to overlap (Masud et al., 2015). In particular, the warmth component of authoritativeness was likely to overlap with the subscale of support and encouragement, and the expectation component was likely to overlap with the subscale of expectations and attitudes. Although we isolated the unique contribution of each construct by including all variables of interest in the same analytic model, whether and how parenting styles and parenting practices can be separately expressed by parents and separately perceived by adolescents awaits further investigation. Qualitative approaches, such as interviews and focus groups, may be particularly helpful in such efforts.

## 5. Conclusions

Despite these limitations, our study has important theoretical and practical implications. At a theoretical level, our findings highlighted the value of conceptualizing both parental educational involvement and adolescent academic adjustment as multidimensional constructs and of distinguishing between parenting practices and parenting styles. At an applied level, our findings pointed to the potential utility of targeting both maternal educational involvement and maternal authoritativeness to promote early adolescents’ academic adjustment.

## Figures and Tables

**Table 1:** Means (*M*) and Standard Deviations (*SD*) of and Correlations Among Variables.

Variables	*M*	*SD*	(2)	(3)	(4)	(5)	(6)	(7)	(8)	(9)	(10)	(11)	(12)
(1) C gender	1.50	0.50	0.07	−0.03	0.04	0.06	0.02	0.02	−0.10 **	−0.09 *	0.02	0.02	−0.12
(2) C age	10.54	0.90		−0.08 *	−0.08	−0.06	0.01	−0.13 **	−0.06	−0.19 **	0.01	0.04	0.06
(3) M education	4.03	1.58			0.12 **	0.06	0.05	0.09 *	0.11 **	0.12 **	0.09 *	0.12 **	0.16 **
(4) M authoritativeness	3.38	0.85				0.78 **	0.47 **	0.36 **	0.31 **	0.25 **	0.49 **	0.41 **	0.30 **
(5) M support and encouragement	2.94	0.78					0.35 **	0.26 **	0.25 **	0.21 **	0.41 **	0.38 **	0.23 **
(6) M expectations and attitudes	4.11	0.89						0.48 **	0.22 **	0.09 *	0.45 **	0.33 **	0.15 **
(7) M rules and monitoring	3.78	0.87							0.34 **	0.23 **	0.35 **	0.31 **	0.10 *
(8) M communication with teachers	2.29	0.80								0.61 **	0.25 **	0.24 **	0.12 *
(9) M participation in school events	1.93	0.73									0.13 **	0.16 **	0.13 **
(10) C academic motivation	3.98	0.68										0.63 **	0.29 **
(11) C academic skills	3.78	0.66											0.43 **
(12) C academic competence	3.24	0.58											

*Notes.* C = Child. M = Mother. * *p* < 0.05. ** *p* < 0.01.

**Table 2:** Standardized Regression Coefficients (*β*) and Variance Explained (Δ*R*^2^) of Models of Early Adolescent Academic Adjustment.

		Motivation		Skills		Competence	
Steps	Variables	Δ*R*^2^	*β*	Δ*R*^2^	*β*	Δ*R*^2^	*β*
(1)	C gender	0.01	0.02	0.02 *	0.02	0.05 *	−0.12 *
	C age		0.02		0.05		0.08
	M education		0.10 *		0.13 *		0.16 *
(2)	M authoritativeness	0.31 *	0.25 *	0.22 *	0.16 *	0.09 *	0.28 *
	M support and encouragement		0.10		0.15 *		0.02
	M expectations and attitudes		0.23 *		0.12 *		0.01
	M rules and monitoring		0.11 *		0.13 *		−0.01
	M communication with teachers		0.07		0.06		−0.02
	M participation in school events		−0.03		0.02		0.05
(3)	Authoritativeness X Expectations	0.02 *	0.16 *	0.02 *	0.09 *	0.03 *	0.12 *
	Authoritativeness X Communication		-		0.12 *		0.12 *

*Notes.* C = Child. M = Mother. * *p* < 0.017.

## Data Availability

Data can be obtained from the corresponding author upon reasonable request.
